# Natural products: potential drugs for the treatment of renal fibrosis

**DOI:** 10.1186/s13020-022-00646-z

**Published:** 2022-08-17

**Authors:** Zijun Zhou, Yanheng Qiao, Yanru Zhao, Xin Chen, Jie Li, Hanqing Zhang, Qiumei Lan, Bo Yang

**Affiliations:** 1 Department of Nephrology, First Teaching Hospital of Tianjin University of Traditional Chinese Medicine, Tianjin, China; 2 Department of Nephrology, National Clinical Research Center for Chinese Medicine Acupuncture and Moxibustion, Tianjin, China

**Keywords:** Renal fibrosis, Inflammation, Oxidative stress, Natural product, Traditional Chinese Herbal Medicine

## Abstract

With the increasing prevalence and mortality, chronic kidney disease (CKD) has become a world public health problem. As the primary pathological manifestation in CKD, renal fibrosis is often used as a critical target for the treatment of CKD and inhibits the progression of CKD to end-stage renal disease (ESRD). As a potential drug, natural products have been confirmed to have the potential as a routine or supplementary therapy for chronic kidney disease, which may target renal fibrosis and act through various pharmacological activities such as anti-inflammatory and anti-oxidation of natural products. This article briefly introduces the pathological mechanism of renal fibrosis and systematically summarizes the latest research on the treatment of renal fibrosis with natural products of Chinese herbal medicines.

## Background

Chronic kidney disease (CKD) has become a world public health problem with the increasing prevalence and mortality. In 2017, the number of patients with CKD reached 697.5 million, and the global prevalence of CKD was 9.1% [1]. At present, the treatment of CKD is mainly based on the use of angiotensin-converting enzyme inhibitors and angiotensin receptor blockers. However, this does not better prevent the progression of CKD [2]. Continuously progressive CKD will eventually develop into end-stage renal disease. At this time, patients can only rely on renal replacement therapy, seriously affecting the quality of life, so the search for better CKD treatment strategies has become a current research hotspot.

The pathological manifestations of CKD due to different causes may vary slightly. However, the main pathological feature is renal fibrosis driven by renal injury stimuli such as inflammation and oxidative stress, so anti-renal fibrosis is widely studied as a potential CKD therapeutic target. Traditional Chinese Medicine (TCM), as an alternative therapy in modern medicine, has attracted much attention in recent years. A large number of studies have demonstrated that natural products in TCM play a role in anti-renal fibrosis through their anti-oxidation and anti-inflammation pharmacological activities.

In this paper, we introduce the pathological mechanism involved in renal fibrosis, summarize the latest research on the treatment of renal fibrosis with natural products in recent years, and discuss the future direction and challenges of natural products of Chinese herbal medicines and renal fibrosis.

## Pathological mechanisms of renal fibrosis

**Fig. 1:**
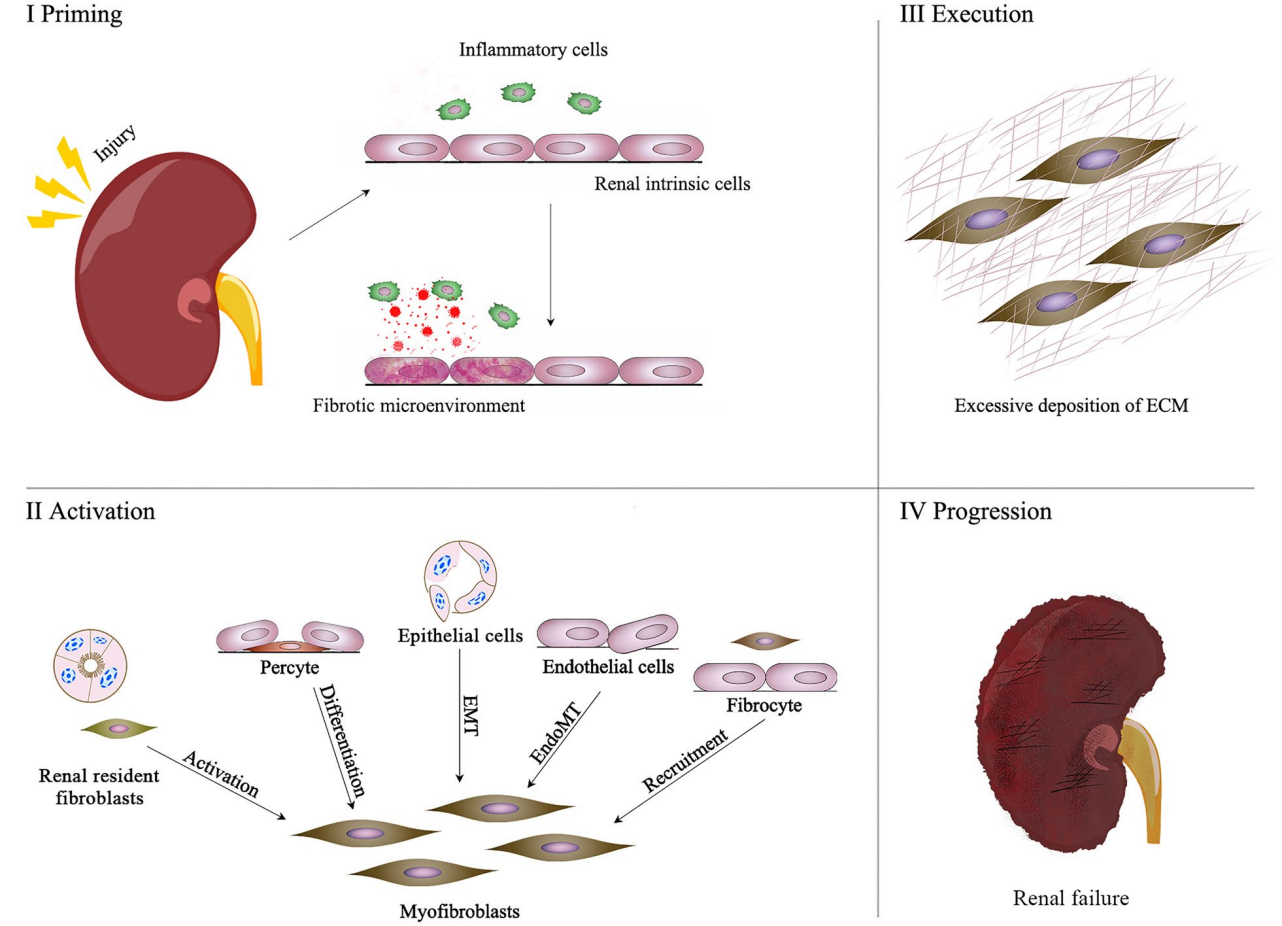
Pathological mechanism of renal fibrosis

Renal fibrosis is the main pathological feature of CKD and plays a vital role in CKD progression to ESRD. The essence of renal fibrosis is that various injury reactions stimulate renal resident cells, causing excessive extracellular matrix (ECM) deposition, tubulointerstitial fibrosis, and glomerulosclerosis, ultimately leading to the destruction of renal parenchyma and loss of renal function [3]. Renal fibrosis involves a series of complex cellular and molecular mechanisms. Almost all renal resident cells are involved in the process of fibrosis. Generally, renal fibrosis can be divided into four overlapping processes: priming, activation, execution, and progression. It is worth noting that these four stages are not strictly chronological. Since fibrosis is a dynamic pathological process, many events may occur simultaneously [4]. This paper will briefly introduce the cellular and molecular pathways involved in these four stages (Fig. 1).

### Priming: formation of the fibrotic microenvironment

In the initiation phase, various renal injurious stimuli such as infection, trauma, inflammation and autoimmunity act on renal resident cells to induce the initiation of fibrosis, the most important of which is the inflammatory response [5]. Inflammation is the most important initiator of renal fibrosis. Under various injury stimuli, inflammatory cells such as lymphocytes, macrophages and dendritic cells are recruited into the glomeruli and renal interstitium. At the same time, these injury stimuli will also activate the resident immune cells of the kidney, produce inflammatory mediators and form an inflammatory microenvironment [6]. Normally, inflammation is conducive to the repair of body injury. However, persistent inflammation is the key cause of initiating fibrosis. Renal resident cells and recruited inflammatory cells stimulated by persistent inflammation release pro-fibrotic cytokines such as inflammatory and growth factors [7] and form a fibrotic microenvironment. The formation of a fibrotic microenvironment promotes the activation and proliferation of myofibroblasts and the imbalance between ECM production and degradation. And then, the process of fibrosis also enters the activation stage.

### Activation: activation of myofibroblasts

Under the stimulation of pro-fibrotic cytokines, matrix-producing cells in the kidney are activated, and fibroblasts, tubular epithelial cells, endothelial cells, podocytes, cells, and macrophages can produce ECM, but usually myofibroblasts are the main effector cells leading to excessive ECM deposition [3]. Myofibroblasts are considered to be a type of cell with both smooth muscle cell and fibroblast characteristics, which are rarely seen in the normal kidney, but are abundant in the fibrotic environment, so the source of myofibroblasts has been a research hotspot and is still controversial. The possible sources are renal resident fibroblasts, pericytes, epithelial cells, endothelial cells and circulating bone marrow-derived fibrocytes, which transform and proliferate into myofibroblasts under the action of pro-fibrotic cytokines. These cytokines also act on myofibroblasts to produce a large amount of ECM and αSMA, which leads to renal fibrosis [8–10].

At this stage, numerous molecular pathways activate myofibroblasts, and the most studied ones are mainly focused on signaling pathways such as TGF-β, Wnt, and Hedgehog, which also play an important role in the next stage. TGF-β is now recognized as the most critical pro-fibrotic factor that can activate myofibroblasts through standard Smad and non-standard MAPK signaling pathways. Wnt/β-catenin can activate myofibroblasts by regulating the expression of downstream genes and can also act by regulating the renin–angiotensin system (RAS). The Hedgehog pathway acts primarily through its ligand Sonic hedgehog (Shh) to regulate the transcription factor Gli. Some reviews have comprehensively summarized the relationship between these pathways and renal fibrosis, so it will not be introduced in detail here [11–14].

### Execution: excessive deposition of ECM

In this stage, matrix-producing cells (especially myofibroblasts) activated by the above pathways begin to synthesize and secrete a large amount of ECM. At the same time, due to the influence of the fibrotic microenvironment, the balance between ECM production and degradation is out of balance, so that they are excessively deposited in glomeruli and renal tubules. This abnormal ECM accumulation will lead to glomerulosclerosis and tubulointerstitial fibrosis [4, 15]. Renal ECM is a non-cellular three-dimensional macromolecular network composed of various glycoproteins such as collagen, elastin, proteoglycan and fibronectin, of which type I and type III collagen and fibronectin play a major role in renal fibrosis, and these proteins play an important role in the process of renal fibrosis under the regulation of integrins and their downstream signals [16, 17].

The abnormal deposition of ECM is mainly because of the imbalance between production and degradation. Among them, the molecular pathways leading to increased ECM production mainly involve two aspects, on the one hand, TGF-β and other signaling pathways activate a large number of myofibroblasts to synthesize and secrete ECM during the activation stage, and on the other hand, these pro-fibrotic signals can directly promote the synthesis and secretion of ECM transcriptionally, in which anti-fibrotic factors (e.g., BMP-7, HGF) can inhibit the production of ECM by antagonizing the TGF-β signaling pathway [3]. The molecular pathways leading to reduced ECM degradation are mainly associated with changes in the expression of metalloproteinases (e.g., MMPs, ADAMs and ADAMTSs, etc.) and metalloproteinase inhibitors (TIMPs) in fibrotic environments [17].

### Progression: progressive renal failure

A large amount of ECM has been deposited in the glomeruli and tubulointerstitium, resulting in the destruction of the original structure and the loss of renal function. At this time, renal fibrosis has entered a vicious cycle, which means that ECM is not only the result after injury, but also can act as a new stimulus to promote fibrosis. This pro-fibrotic effect may be related to the synergistic regulation of the YAP/TAZ and TGF-β signaling pathway [18]. In addition to the effects of ECM on renal function, since numerous pro-fibrotic factors similarly involve inflammation [19], oxidative stress [20], autophagy [21], and other signaling pathways, these cellular and molecular events also damage renal resident cells while promoting fibrosis, which can also lead to further loss of renal function. The progressive stage is the final stage of renal fibrosis, during which renal function continues to decline until ESRD is entered.

## Therapeutic effects of natural products on renal fibrosis

Natural products have been considered as one of the essential sources for drug research and development, and in fact, 441 natural products and their derivatives were approved by the FDA for clinical use as drugs in the course of 1981 to 2019 [22]. Of the 371 medicinal substances included in the Ninth Edition of International Pharmacopoeia, more than 80 are natural products and their derivatives [23]. In the related research of renal fibrosis, a large number of natural products (especially the natural products in Traditional Chinese Medicine) have been confirmed to alleviate the process of renal fibrosis, protect the renal structure and improve renal function by regulating a variety of cytokines. This paper is divided into the following categories according to their different chemical structures and systematically summarizes the mechanism of action of natural products in Traditional Chinese Herbal Medicine in protecting renal fibrosis [24] (Table 1).

**Table 1:** Natural products and renal fibrosis

Natural products	Structure	Model	Signaling pathway	Refs.
Quercetin		UUO mice Mouse macrophage line cells	M1/M2 macrophage polarization	[26]
		UUO rats NRK-52E cells	Sonic Hedgehog pathway	[27]
		UUO rats NRK-52E cells	SIRT1/PINK1/Parkin pathway	[28]
Puerarin		UUO mice HK-2 cells	Oxidative stress MAPK pathway	[29]
		UUO mice	NF-κB p65/STAT3 pathway TGF-β/Smad pathway	[30]
Dihydromyricetin		UUO mice HK-2 cells	miR-34a pathway	[31]
		Human glomerular mesangial cells	Nrf2/HO-1 pathway	[33]
Calycosin		DN rats	IL-33/ST2 pathway	[34]
Isoliquiritigenin		Mouse bone marrow-derived macrophage UUO mice	Mincle/Syk/NF-κB pathway M1 macrophage polarization inflammation	[34]
		NRK-52E cells DN mice	SIRT1 pathway Inflammation Oxidative stress	[35]
		HK-2 cells UUO mice	Cellular senescence	[36]
5,7,3ʹ,4ʹ,5ʹ-pentahydroxy flavanone		5/6 nephrectomized rats NRK-52E cells	AHR pathway	[37]
Barleriside A		5/6 nephrectomized rats NRK-52E cells	AHR pathway	[37]
Rhoifolin		5/6 nephrectomized rats NRK-52E cells	AHR pathway	[37]
5,6,7,8,3ʹ,4ʹ-hexa-methoxyflavone		5/6 nephrectomized rats HK-2 cells	AHR pathway	[38]
Curcumin		UUO rats	NLRP3 inflammasome PI3K/AKT/mTOR pathway	[42]
		Kidney transplantation rats	Autophagy	[43]
		Cisplatin-induced rats	Oxidative stress	[44]
Resveratrol		UUO rats	MAPK pathway PI3K/AKT pathway Wnt/β-catenin pathway JAK2/STAT3 pathway	[45]
		Aged mice	Renin–angiotensin System	[46]
		Concanavalin A-induced Aged mice	SIRT1/Klotho pathway Oxidative stress	[47]
Epigallocatechin gallate		Cadmium-induced rats	TGF-β1/microRNA pathway Oxidative stress	[48]
		Dahl salt-sensitive rats NRK-49F cells	Inflammation Oxidative stress Apoptosis	[49]
		DN mice HEK293 cells	Notch pathway TGF-β/Smad3 pathway	[50]
Salvianolic acid A		5/6 nephrectomized rats HK-2 cells	p38 MAPK/NF-κB	[51]
Schisandrin B		UUO mice HK-2 cells NRK-52E cells HEK-293 T cells	TGF-β1 pathway miR-30e pathway	[52]
Poricoic acid A		UUO mice NRK-52E cells NRK-49F cells	TPH-1 pathway Wnt/β-catenin pathway	[56]
		5/6 nephrectomized rats UUO rats NRK-49F cells	TGF-β1/Smad3 pathway AMPK pathway	[57]
Poricoic acid ZC		UUO mice HK-2 cells	TGF-β/Smad pathway Wnt/β-catenin pathway	[58]
Poricoic acid ZD		UUO mice HK-2 cells	TGF-β/Smad pathway Wnt/β-catenin pathway	[58]
Poricoic acid ZE		UUO mice HK-2 cells	TGF-β/Smad pathway Wnt/β-catenin pathway	[58]
Poricoic acid ZG		HK-2 cells	TGF-β/Smad pathway Wnt/β-catenin pathway	[59]
Poricoic acid ZH		HK-2 cells	TGF-β/Smad pathway Wnt/β-catenin pathway	[59]
Poricoic acid ZI		UUO mice HK-2 cells NRK-52E cells NRK-49F cells	TGF-β/Smad pathway Wnt/β-catenin pathway	[60]
Poricoic acid ZM		UUO mice HK-2 cells	NF-κB pathway Keap1/Nrf2 pathway AHR pathway	[61]
Poricoic acid ZP		UUO mice HK-2 cells	NF-κB pathway Keap1/Nrf2 pathway AHR pathway	[61]
Alisol B 23-acetate		5/6 nephrectomized rats UUO rats NRK-52E cells NRK-49F cells	Gut–kidney axis RAS TGF-β/Smad pathway Wnt/β-catenin pathway	[62]
Triptolide		Col4a3^–/–^ mice DN mice Folic acid-induced mice HK-2 cells	PTEN pathway	[63]
		DN rats Human mesangial cells	miR-141-3p/PTEN/Akt/mTOR pathway	[64]
Ligustrazine		UUO rats	TGF-β1/CTGF pathway HGF pathway	[67]
Oxymatrine		NRK-52E cells	SnoN pathway	[68]
		db/db mice NRK-52E cells	Id2 pathway Twist pathway	[69]
Leonurine		UUO mice	TGF-β pathway NF-κB pathway	[70]
Berberine		DN mice NRK-52E cells	Nrf2/HO-1 pathway TGF-β/Smad pathway	[71]
		DN mice Mouse renal tubular epithelial cells	Notch/snail pathway	[72]
Astragaloside IV		DN rats	TRX antioxidant system NLR pathway	[74]
		UUO mice	TLR4/NF-κB pathway	[75]
		HK-2 cells	mTORC1/p70S6K pathway	[76]
		Tacrolimus-induced mice	p62-Keap1-Nrf2 pathway	[77]
Salidroside		UUO mice Folic acid-induced mice HK-2 cells	TLR4/MAPK/NF-κB pathway	[78]
		DN mice	SIRT1/PGC-1α pathway	[79]
		Adriamycin‐induced mice	Wnt/β-catenin pathway	[80]
Dioscin		Fructose-induced rats	SIRT3 pathway TGF-β1/Smad3 pathway	[81]
Tanshinone IIA		5/6 nephrectomized rats	TGF-β/Smad pathway NF-κB pathway	[84]
		Folic acid-induced mice IRI mice	GSK3β pathway MAPK pathway	[85]
		DN rats	PERK pathway Oxidative stress	[86]
Emodin		DN rats	AMPK/mTOR pathway	[87]
		Adriamycin-induced rats with unilateral nephrectomy HK-2 cells	BMP7/TGF-β1 pathway Autophagy	[88]
		UUO rats NRK-49F cells	EZH2 pathway	[89]
Chrysophanol		UUO mice HK-2 cells	TGF-β/Smad pathway	[90]

### Flavonoids

Flavonoids are widely present in a variety of Chinese herbal medicines and are common natural products, which have various biological activities such as regulating oxidative stress, participating in cell cycle arrest, inducing apoptosis, autophagy, and so on [25]. In recent years, the anti-fibrotic effects of some flavonoids have become a research hotspot.

Quercetin is a natural flavonoid, which exists in many kinds of Chinese herbs and has many pharmacological effects, such as anti-inflammatory and anti-oxidation. Quercetin has been found to inhibit the expression of NF-κB p65 and IRF5 signaling pathways in the kidneys of UUO mice, which in turn inhibit M1 macrophage polarization and the expression of inflammatory factors and treat kidney injury. At the same time, it can reduce the expression of NF-κB p50 and IRF4 signaling pathways, inhibit M2 macrophage polarization, which reduces the deposition of ECM and alleviate renal interstitial fibrosis [26]. Liu et al. found that quercetin can also inhibit the expression of SHH signaling in the kidneys of UUO rats, prevent EMT in tubular epithelial cells, reduce excessive accumulation of ECM, and treat renal fibrosis [27]. In addition, it has also been found that quercetin can inhibit tubular epithelial cell senescence and reduce renal fibrosis by activating SIRT1/PINK1/Parkin-mediated mitosis [28].

Puerarin, a natural product extracted from Radix Puerariae, has been found to have an anti-fibrotic effect in recent years, and Zhou et al. found that puerarin can inhibit oxidative stress-induced tubular epithelial cell apoptosis and improve renal fibrosis by decreasing ROS production and the expression of MAPK signaling pathways in the kidneys of UUO mice [29]. Others have found that puerarin can reduce fibrosis by inhibiting the NF-κB p65/STAT3 and TGF-β/Smad signaling pathways and inhibiting the inflammation and excessive deposition of ECM in the kidney [30].

Dihydromyricetin is mainly derived from Chinese herbal medicines such as Ampelopsis Japonica and has a wide range of pharmacological activities. In the UUO mice model, dihydromyricetin inhibited TGF-β1-mediated miR-34a expression in the kidney, which up-regulated Klotho expression in tubular epithelial cells and alleviated renal fibrosis [31]. In high glucose-induced glomerular cells, dihydromyricetin can also improve renal fibrosis by regulating the Nrf2/HO-1 signaling pathway and inhibiting the deposition of ECM and the expression of fibronectin [32].

Calycosin is the main component of Astragalus membranaceus, and recent studies have shown that calycosin can improve the inflammatory response and fibrosis in diabetic nephropathy and protect the renal structure by inhibiting the expression of inflammatory mediators IL-33/ST2 signaling pathway and its downstream inflammatory factors [33].

Isoliquiritigenin is a natural flavonoid from Glycyrrhiza uralensis and has anti-fibrotic effects. Studies have shown that isoliquiritigenin directly inhibits the Mincle/Syk/NF-κB signaling pathway in UUO mice while inhibiting the polarization of M1 macrophages and reducing renal inflammation and fibrosis [34]. In addition, isoliquiritigenin also has a good therapeutic effect on kidney injury in diabetic nephropathy and treats renal fibrosis by regulating oxidative stress and inflammation mediated by the SIRT1 pathway [35]. Isoliquiritigenin can also inhibit the expression of ITGB3, ameliorate tubular cell senescence, and reduce renal fibrosis caused by senescence in the kidney [36].

5,7,3ʹ,4ʹ,5ʹ-pentahydroxy flavanone, Barleriside A, and Rhoifolin are natural flavonoids derived from Semen Plantaginis. 5,6,7,8,3ʹ,4ʹ-hexamethoxyflavone is a natural flavonoid derived from Poria cocos. Although they have different structures, they are all aryl hydrocarbon receptor (AHR) antagonists. In 5/6 nephrectomy rat models, they significantly reduced the secretion of ECM by regulating the aromatic hydrocarbon receptor signaling pathway, while inhibiting EMT of epithelial cells and alleviating renal fibrosis [37, 38].

### Polyphenols

Polyphenols, also known as polyhydroxyphenols, have anti-inflammatory and anti-oxidation pharmacological effects, but also can regulate immunity and cell proliferation, and have a good therapeutic effect on various chronic inflammatory diseases [39–41]. Polyphenolic compounds have therefore also attracted much attention in the field of anti-fibrosis.

Curcumin is the main active component in Curcumaelongae Rhizoma, which has been demonstrated to have an excellent anti-fibrotic effect. It was found that mitochondrial dysfunction was significantly improved in renal interstitial cells of UUO rats after curcumin treatment, which in turn inhibited the activation of NLRP3 inflammasome and the expression of PI3K/AKT/mTOR signaling pathway, alleviating the progression of renal fibrosis by reducing the inflammatory response and regulating autophagy [42]. Curcumin can also attenuate EndMT and fibrosis occurring after kidney transplantation, which is similarly accomplished by activating cellular autophagy [43]. In addition, curcumin can act as an anti-oxidant that can alleviate renal fibrosis induced by scavenging excess ROS, inhibiting the activity of NADPH oxidase, improving mitochondrial redox balance [44].

Resveratrol is mainly derived from plants such as Cassiae Semen and Polygoni Cuspidati Rhizoma Et Radix and can also be obtained in plants such as grapes and peanuts, which are widely used in traditional medicines and dietary supplements. Resveratrol has been found to inhibit tubular epithelial cell EMT and fibroblast proliferation and differentiation, prevent myofibroblasts’ activation and improve renal fibrosis by inhibiting the activity of proliferation-related signaling pathways of tubular epithelial cells and interstitial cells [45]. In addition, resveratrol has been found to reduce renal oxidative stress and delay glomerulosclerosis and renal interstitial fibrosis in the aging kidney by regulating the renin–angiotensin system [46]. Chen et al. found that resveratrol could up-regulate SIRT1-mediated Klotho expression and the expression of anti-oxidant factors such as SOD and GSH and ameliorate progressive glomerulosclerosis in aging kidneys [47].

Epigallocatechin gallate (EGCG) is the most important polyphenolic compound in green tea and has an excellent protective effect on kidney injury caused by various causes. For chronic kidney injury due to cadmium intoxication, EGCG can ameliorate renal fibrosis by regulating the expression of TGF-β1 and its mediated microRNAs, restoring anti-oxidation enzymes activity in renal cells, inhibiting EMT and reducing the excessive deposition of ECM in renal cells [48]. In renal injury caused by salt-sensitive hypertension, EGCG reduces renal cellular inflammatory infiltration and oxidative stress, improves renal injury through anti-inflammatory and anti-oxidation effects, and improves renal fibrosis by inducing fibroblast apoptosis [49]. In diabetic nephropathy, EGCG can inhibit the expression of the TGF-β/Smad3 signaling pathway by binding with Notch1, attenuating fibrosis [50].

Salvianolic acid A is a natural product derived from Radix Salviae. In 5/6 nephrectomy rats model, salvianolic acid A significantly reduced the expression of p38 MAPK and its downstream signal inflammatory factors such as NF-κB, while inhibiting the expression of TGF-β1 and α-SMA in renal cells, reducing renal inflammation and renal interstitial fibrosis, and exerting a protective effect on the kidney [51].

Schisandrin B is mainly derived from the traditional Chinese medicine Schisandrae Chinensis Fructus, which has been found to inhibit the expression of Snail, Slug and Zeb2, preventing EMT in tubular epithelial cells, and reduce TGF-β1-mediated renal interstitial fibrosis by up-regulating the expression of miR-30e in renal cells [52].

### Terpenoids

Terpenoids are important natural products in Chinese herbal medicines, which have many potential pharmacological activities such as anti-cancer, anti-fibrosis, anti-inflammatory, etc. [53–55]. As a potential drug, many studies have reported on the anti-fibrosis effects of terpenoids.

Poricoic acid A is one of the main active ingredients in Poria cocos and has an excellent anti-renal fibrosis effect. In the UUO mice model, poricoic acid A reduced the activity of the Wnt/β-catenin signaling pathway by enhancing the expression of tryptophan hydroxylase-1 (TPH-1), and also inhibited renal cell injury and fibroblast activation, exerting an anti-renal fibrosis effect [56]. In addition, poricoic acid A can also inhibit renal fibrosis by activating the AMPK signaling pathway to inhibit TGF-β1/Smad3 pathway-mediated deposition of ECM and activation of myofibroblasts [57].

Poricoic acid ZC, ZD, ZE, ZG, ZH, ZI, ZM, and ZP are novel tetracyclic triterpenoid compounds newly discovered in recent years, which are the main components of Poria cocos, and have renoprotective effects. Among them, Poricoic acid ZC, ZD, ZE, ZG, and ZH significantly ameliorate renal tubular interstitial fibrosis by inhibiting TGF-β/Smad and Wnt/β-catenin signaling pathways [58, 59]. Poricoic acid ZI reduces the secretion of ECM and attenuates epithelial cells EMT by inhibiting the activity of MMP-13 [60]. Poricoic acid ZM, ZP inhibits the expression of NF-κB and its downstream genes, promotes the expression of the Nrf2 signaling pathway, regulates AHR signaling pathway, attenuates oxidative stress and inflammatory response in the kidney, and treats renal fibrosis [61].

Alisol B23-acetate is a triterpenoid derived from Alisma Orientale. Chen et al. found that alisol B23-acetate could reduce renal fibrosis in UUO rats and 5/6 nephrectomy rats, which may be associated with improving gut microbiota and then reducing blood pressure and regulating the RAS. In addition, alisol B23-acetate can also inhibit the activation of Smad3 and the activation of the Wnt/β-catenin signaling pathway, induce fibroblast apoptosis and inhibit their activation and proliferation, reduce renal interstitial fibrosis [62].

Triptolide is mainly derived from Tripterygii Radix and has good efficacy in various kidney diseases. Studies have found that triptolide can specifically bind to MEX3C protein in the kidney and inhibit MEX3C-mediated K27-linked polyubiquitin chain modification of phosphatase and tensin homolog, thereby inhibiting EMT in tubular epithelial cells and protecting renal function [63]. In particular, in diabetic nephropathy models, triptolide can also restore autophagy in glomerular fibrotic cells by regulating the miR-141-3p/PTEN/Akt/mTOR signaling pathway to reduce fibrosis [64].

### Alkaloids

Alkaloids have many pharmacological activities, such as anti-inflammatory, anti-oxidation, and anti-cancer. They are one of the natural sources of drugs and are the active ingredients of many kinds of traditional Chinese medicines [55, 65, 66]. At present, the research on the anti-fibrosis of alkaloids has become a current research hotspot.

Ligustrazine is a natural product of Chuanxiong Rhizoma and is mainly used to treat various kidney injuries. Yuan et al. showed that ligustrazine decreased the expression of TGF-β1 and CTGF, up-regulated the expression of HGF and BMP-7 in tubular epithelial cells, and inhibited EMT in tubular epithelial cells to alleviate renal interstitial fibrosis [67].

Oxymatrine can be mainly found in Sophorae Flavescentis Radix and has been demonstrated to have anti-organ fibrosis effects. Liu et al. found in vitro that oxymatrine could inhibit TGF-β1/Smad-mediated EMT in epithelial cells by up-regulating the expression of nuclear transcription co-repressor Ski-related novel protein N [68]. In addition, it has been found that in diabetic nephropathy mice treated with oxymatrine, the expression of inhibitor of differentiation 2 (Id2) was significantly increased in the kidney, which suggests that oxymatrine may play a role in anti-renal fibrosis by restoring the expression of Id2 and promoting the binding of Id2 and Twist in the damaged kidney thereby regulating the expression of genes downstream of Twist and inhibiting EMT in tubular epithelial cells [69].

Leonurine is an active component in Leonuri Herba and has pharmacological activities of anti-inflammatory and anti-oxidation. In UUO mice, leonurine ameliorates inflammation and renal interstitial fibrosis in the kidney by inhibiting the ROS-mediated NF-κB signaling pathway and TGF-β/Smad3 signaling pathway [70].

Berberine is a natural product of Chinese herbal medicine such as Coptidis Rhizoma and Phellodendri Chinrnsis Cortex, widely used in clinical practice. Berberine has been found to inhibit the expression of the TGF-β/Smad pathway while promoting the Nrf2/HO-1 pathway, preventing EMT and excessive accumulation of ECM in tubular epithelial cells and alleviating renal fibrosis [71]. In addition, berberine also inhibits Notch/snail expression in tubular epithelial cells and prevents EMT progression and renal interstitial fibrosis [72].

### Glycosides

Glycosides are the active ingredients of many kinds of traditional Chinese medicines, which have many potential pharmacological activities and also have good efficacy in anti-inflammatory and anti-fibrosis [55, 73].

Astragaloside IV (AS-IV) is a natural product in Astragalus membranaceus, which has a good renoprotective effect and can improve renal fibrosis mainly through anti-inflammatory and anti-oxidative stress. Zhang et al.’s study found that AS-IV could significantly up-regulate the expression of TRX1, decrease the expression of cytokines such as TXNIP, PANX1, NOD2, and JUN in the kidneys of DN rats, inhibit inflammation-related NLR signaling pathway expression by enhancing the TRX anti-oxidant system, and attenuates renal injury, fibrosis, and microstructural changes induced by diabetic nephropathy [74]. Zhou et al. found that AS-IV can attenuate inflammation and inhibit renal fibrosis by inhibiting TLR4/NF-κB signaling pathway [75]. In vitro experiments have shown that AS-IV can also inhibit EMT in tubular epithelial cells and ameliorate renal fibrosis by inhibiting the mTORC1/p70S6K signaling pathway [76]. In addition, AS-IV also has a good therapeutic effect in kidney injury induced by some nephrotoxic drugs. In a study on tacrolimus-induced chronic nephrotoxicity, AS-IV was found to reduce ROS accumulation and renal interstitial fibrosis by regulating the p62-Keap1-Nrf2 signaling pathway [77].

Salidroside is the main component of Rhodiola Rosea, which has the function of protecting the kidney. Studies have confirmed that salidroside reduces excessive deposition of ECM, prevents epithelial cell EMT, and ameliorates renal fibrosis by inhibiting the expression of TLR4/MAPK/NF-κB signaling pathway and its downstream pro-inflammatory and pro-fibrotic factors [78]. Salidroside can also regulate the SIRT1/PGC-1α signaling pathway to improve mitochondrial dysfunction, reduce renal fibrosis in diabetic nephropathy, and protect renal function [79]. Salidroside can also modulate Wnt/β-catenin signaling in a model of adriamycin-induced nephropathy that alleviates podocyte injury and renal fibrosis [80].

Dioscin, a natural product in Rhizoma Dioscoreae, has been found to up-regulate the expression of the SIRT3 gene, inhibit renal fibrosis mediated by TGF-β1/Smad3 signaling pathway, and ameliorate fructose-induced kidney injury [81].

### Quinones

Quinones are natural products widely distributed in a variety of traditional Chinese medicines and have been reported to have various pharmacological activities such as antimalarial and anti-tumor activities, and quinones also play an important role in anti-fibrosis [82, 83].

Tanshinone IIA is mainly derived from Radix Salviae and has a significant therapeutic effect on various acute and chronic kidney injuries. Tanshinone IIA significantly reduced excessive deposition of ECM and inflammatory cell infiltration, inhibited renal fibrosis and renal inflammation, and protected renal function by regulating the expression of TGF-β/Smad and NF-κB signaling pathways in 5/6 nephrectomized rats [84]. In folic acid-induced acute kidney injury, tanshinone IIA attenuates tubular inflammatory infiltration and improves renal interstitial fibrosis by inhibiting the excessive activation of GSK3β and subsequent excessive activation of the MAPK pathway [85]. In addition, Xu et al. found that tanshinone IIA can also alleviate oxidative stress status by increasing SOD activity, which inhibits ER stress mediated by the PERK pathway and reduces the expression of TGF-β1, and ameliorates renal fibrosis caused by diabetic nephropathy [86].

Emodin is a natural product in Chinese herbal medicine such as Rheum Offcinale and Polygoni Cuspidati Rhizoma Et Radix, which has anti-fibrotic pharmacological effects. Emodin has been found to reduce renal fibrosis in DN rats by regulating the AMPK/mTOR signaling pathway in the kidney, promoting podocyte autophagy, and inhibiting apoptosis [87]. Emodin can also improve renal interstitial fibrosis by up-regulating the expression of BMP7 and promoting autophagy in tubular epithelial cells and inhibiting their EMT [88]. In addition, in the UUO rats model, emodin inhibited the expression of enhancer of zeste homolog 2, which in turn inhibited trimethylation on Lysine 27 of histone H3 and alleviated the process of tubulointerstitial fibrosis [89].

Chrysophanol is a natural anthraquinone compound in Rheum Officinale with a variety of pharmacological activities. It was found that Chrysophanol alleviated renal fibrosis in UUO mice by modulating the TGF-β/Smad signaling pathway, especially inhibiting phosphorylation of Smad3 [90].

## Conclusion

The incidence and mortality of CKD are increasing yearly worldwide, and renal fibrosis, as the primary pathological manifestation of CKD, has been a targeted therapeutic target. Natural products in Chinese herbal medicine perform well in the process of anti-renal fibrosis due to their anti-oxidation and anti-inflammation pharmacological effects. This review comprehensively summarizes the therapeutic effects and the molecular mechanisms of natural products in Chinese herbal medicine on renal fibrosis in recent years. These studies have shown that natural products have great potential in anti-fibrosis and are promising as novel therapeutic drugs for CKD.

However, some issues deserve our consideration: First, these studies are based on animal experiments and cell experiments, they are not enough to support the clinical application of these natural products, and we should identify natural products with a precise mechanism of action based on high-quality studies further to confirm the safety and effectiveness of clinical efficacy. Second, existing studies mainly focus on inflammation and oxidative stress, TGF-β/Smad, and Wnt/β-catenin signaling pathways, which have limitations and lack the diversity of therapeutic targets, so more studies are needed to explore other cellular and molecular pathways that may be involved. Third, the effects of the kinetics and pharmacodynamics of natural products on the treatment of renal fibrosis should also be considered. For example, emodin has been shown to have an anti-fibrotic effect in animal experiments and in vitro experiments. However, its poor oral availability may affect clinical efficacy [91]. Finally, there is also a relatively interesting question, whether it needs to rely on the guidance of TCM theory in the search for natural products in Chinese herbal medicines to treat renal fibrosis, because some natural products may not be commonly used drugs in TCM to treat kidney disease, but they have been shown to have an anti-fibrosis effect on other organs, and whether this anti-fibrosis effect is also applicable in the kidney is also a question worth pondering.

In conclusion, this review introduces the pathological processes involved in renal fibrosis, systematically summarizes the latest research on the treatment of renal fibrosis with natural products of Chinese herbal medicines, and points out the problems that need attention in future research, hoping that this paper can provide help for further research in the future.

## Data Availability

All the data used to support the findings of this study are available from the corresponding author upon reasonable request.

## Abbreviations

AS-IV: Astragaloside IV
CKD: Chronic kidney disease
ECM: Extracellular matrix
EGCG: Epigallocatechin gallate
EMT: Epithelial–mesenchymal transition
ESRD: End-stage renal disease
RAS: Renin–angiotensin system
TCM: Traditional Chinese medicine
