# Correction: Natural products: potential drugs for the treatment of renal fibrosis

**DOI:** 10.1186/s13020-022-00668-7

**Published:** 2022-10-24

**Authors:** Zijun Zhou, Yanheng Qiao, Yanru Zhao, Xin Chen, Jie Li, Hanqing Zhang, Qiumei Lan, Bo Yang

**Affiliations:** 1 Department of Nephrology, First Teaching Hospital of Tianjin University of Traditional Chinese Medicine, Tianjin, China; 2 Department of Nephrology, National Clinical Research Center for Chinese Medicine Acupuncture and Moxibustion, Tianjin, China

## Correction to: Chinese Medicine (2022) 17:98 https://doi.org/10.1186/s13020-022-00646-z

Following publication of the original article [1], the authors reported that the sentence below was missing in the article.

Zijun Zhou and Yanheng Qiao contributed equally to this work.

The correct information has been provided in this Correction.

The original article [1] has been corrected.
